# Effects of UV for Cycloaliphatic Epoxy Resin via Thermokinetic Models, Novel Calorimetric Technology, and Thermogravimetric Analysis

**DOI:** 10.1038/s41598-018-34181-5

**Published:** 2018-10-26

**Authors:** Bin Laiwang, Shang-Hao Liu, Yun-Ting Tsai, Jun Deng, Hui-Chun Jiang, Bei Li, Chi-Min Shu

**Affiliations:** 1 School of Chemical Engineering, Anhui University of Science and Technology, Anhui, 232001 PR China; 2 Doctoral Program, Graduate School of Engineering Science and Technology, National Yunlin University of Science and Technology (YunTech), Yunlin, 64002 Taiwan Republic of China; 3 School of Chemical Engineering and Technology, Xi’an Jiao Tong University, Xi’an, 710049 Shaanxi PR China; 4 College of Safety Science and Engineering, Key Laboratory of Western Mine Exploitation and Hazard Prevention of Ministry of Education, Xi’an University of Science and Technology, Xi’an, 710054 Shaanxi PR China; 5 College of Safety Science and Engineering, Nanjing Tech University, Nanjing, 210009 Jiangsu PR China; 6 School Chemical Machinery & Safety Engineering, Dalian, 116024 Liaoning PR China; 7 Center for Process Safety and Industrial Disaster Prevention, School of Engineering, YunTech, Yunlin 64002 Taiwan, Republic of China

## Abstract

The cycloaliphatic epoxy resin selected for this study was 3,4-epoxycyclohexane methyl-3′4′-epoxycyclohexyl-carboxylate (EEC). Epoxy resin has numerous applications, such as varnishes, tires, and electronic materials. However, the extensive used of chlorofluorocarbon (CFC) compounds in the last century has resulted in the formation of a hole in the ozone layer. As a consequence, solar radiation is intensifying gradually; therefore, continuous irradiation by sunlight should be avoided. The results of solar radiation can exacerbate the deterioration and photolysis of compounds. Through thermogravimetry and differential scanning calorimetry, the apparent onset temperature of EEC and EEC was analyzed under UV radiation for different durations. Thermokinetic data were used to determine the parameters of thermal decomposition characteristics through simulation to assess the reaction of EEC and EEC under UV radiation for different durations. The goal of the study was to establish the parameters of thermal decomposition characteristics for the effects of UV on EEC, as well as the probability of severity of thermal catastrophe.

## Introduction

Chemical industries are inseparably connected to people’s livelihood, and chemical products facilitate people’s lives more conveniently. When people enjoy the achievements of chemical products, they normally do not perceive that the chemical product’s properties may cause environmental contamination, disastrous cases, social impacts, and so on.

One of the common chemical products principally used in the world is epoxy resin^1–4^. Epoxy resin has numerous applications, such as tire processing, electronic materials, motherboard, paints, and other industrial dispositions^5–11^. However, the extensive use of chlorofluorocarbon (CFC) compounds in the last century has resulted in the formation of holes in the ozone layer. As a consequence, solar radiation is intensifying gradually. The results of solar radiation may exacerbate the deterioration and photolysis of numerous compounds. Previous studies have shown that ultraviolet (UV) radiation causes the decomposition by light of some materials, such as plastic, dye, and rubber^12–15^. After the materials undergo photolysis, they deteriorate and are broken down into smaller compounds that are harmful to people. Hence, the aim of this study was to determine the effects of UV on cycloaliphatic epoxy resin^16,17^. The target material was 3,4-epoxycyclohexane methyl-3′4′-epoxycyclohexyl-carboxylate (EEC).

We explored and confirmed the thermal decomposition characteristics of EEC, applying thermogravimetry (TG) and differential scanning calorimetry (DSC) to obtain a series of thermal parameters, including mass loss (%), mass loss derivative (%/min), apparent onset temperature (*T*_0_), heat of decomposition (Δ*H*_d_), and peak temperature (*T*_p_). We then combined the TG data with thermokinetics to obtain simulation data under various scenarios. Finally, the simulation results were compared with experimental data for the purpose of loss prevention protocol.

## Methodology

### Sample

The sample used in this study was 99.0% by mass of EEC that was produced by Chang Chun Petrochemical Group, Taiwan, ROC. The physical properties of EEC are listed in Table 1^18^. Since its viscosity is higher than normal liquids, it needed to be saved in a sealed environment. As planned, the controlled factor was UVA (312 nm), which was 18 W (East Lighting Co., Taiwan, ROC). UV was a vital factor applied in the experiment, and the durations of UV radiation were one and two months.

**Table 1:** Physical properties parameters of EEC^16^.

Chemical name	Molecular formula	CAS number	Structure
3,4-Epoxycyclohexylmethyl-3′,4′-epoxycyclo-hexane carboxylate	C_14_H_20_O_4_	2386-87-0	
Mass%	Density (g/cm^3^)	Viscosity (mpa s)	MP (°C)
99.0	1.17	400.0	−37.0

### Thermogravimetry (TG)

TG was used to determine the selected substances with the reduction or increase of decomposition, oxidation, or volatilization (e.g., moisture). The most common applications of TG were included in the analysis of the properties of substances by decomposition mode, the study of degradation mechanisms and reaction kinetics, the determination of organic matter contents in samples, and the determination of inorganic matter (ash) in the samples, which might be used to accurately predict the structure as a chemical analysis^19–22^. Hence, this study deliberately selected PerkinElmer Pyris 1 TG as the instrument to measure the samples. The test temperature ranged from 30.0 to 550.0 °C. The heating conditions were 1.0, 2.0, 4.0, and 8.0 °C/min, the carrier gas in TG was air, and the flow rate was 20.0 mL/min.

### Differential Scanning Calorimetry (DSC)

DSC^23^ is a common thermal calorimeter for measuring heat generation from a sample. It can be used to determine the exothermic and endothermic reactions from its thermal curves. Furthermore, it can acquire some specific thermal decomposition characteristics and process safety parameters for calculating and simulating thermal hazards by kinetic models. DSC is a powerful instrument that has numerous applications, such as in medicine, materials, membranes, liquid crystal, and even explosives. The Mettler Toledo 821^e^ DSC’s mode chosen was non-isothermal for this study. The heating rate of this test was set at 1.0 °C/min. The heating range was 30.0–450.0 °C and the carrier gas was air.

### Gas Chromatography–Mass Spectrometry (GC-MS)

GC-MS^24,25^ is one of the commonly used instruments for identifying and separating organic molecules. It is a typical chemical analysis method for accurate quantitative and qualitative analysis. In this case, GC-MS was used to observe and compare the EEC after pure EEC and UV light. The experiments were performed using a PerkinElmer Clarus 680 gas chromatograph (GC) connected to a PerkinElmer Clarus 600T mass spectrometry (MS). The relative affinity of the stationary phases of the different molecules in the sample would promote the separation of the molecules. The column used in the GC experiment was Elite-624, which had a length and an inner diameter of 30.0 m × 0.25 mm and a film thickness of 1.4 μm. The molecular effluent of the column was captured, and the electron ionization (70 eV) fragment was detected using their mass to charge ratio. A helium flow rate of 1.0 mL/min was used as the carrier gas for the column.

## Results and Discussion

TG and DSC were selected to test EEC, in order to understand the properties of EEC; the functional information is listed in Table 1.

### Nonisothermal TG Test

**Figure 1:**
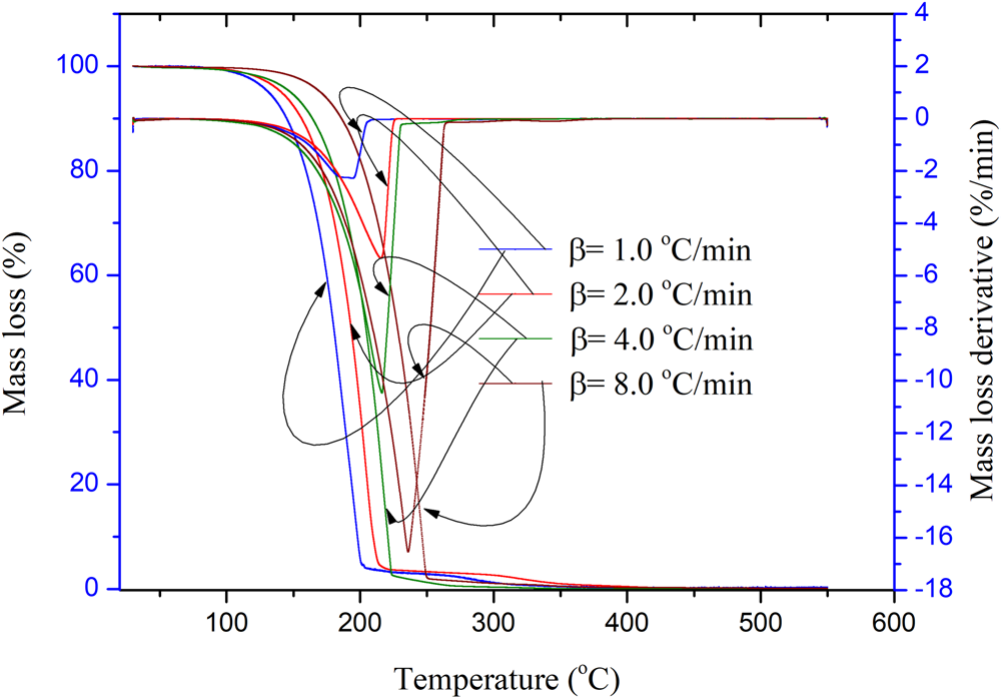
TG and DTG experimental results of EEC at four heating rates of 1.0, 2.0, 4.0, and 8.0 °C/min.

**Figure 2:**
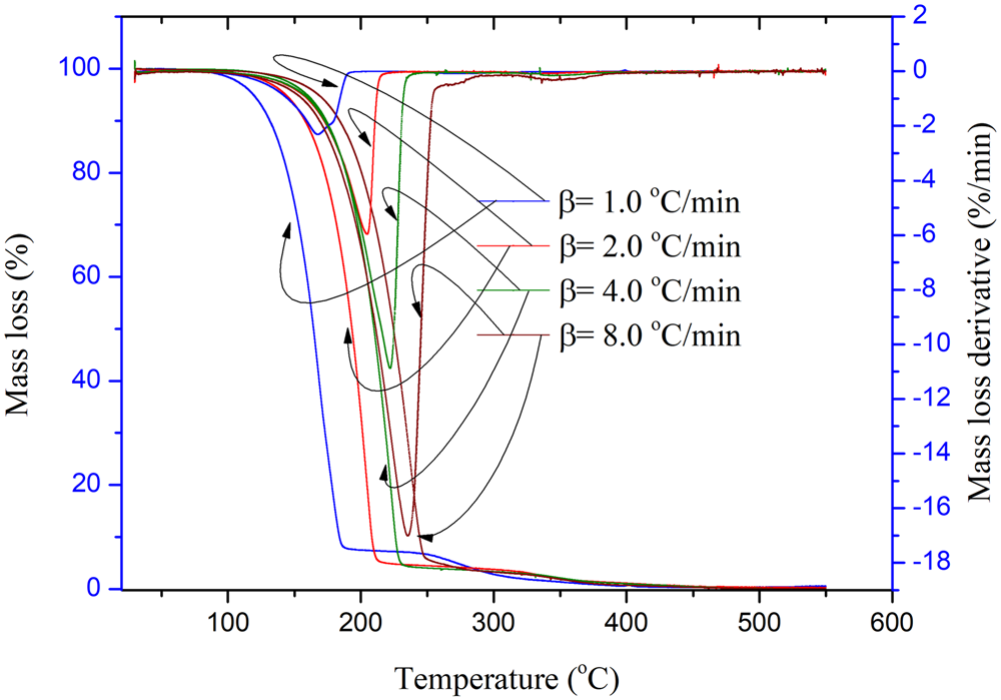
TG and DTG experimental results of EEC under prominent UV radiation for one month at four heating rates of 1.0, 2.0, 4.0, and 8.0 °C/min.

**Figure 3:**
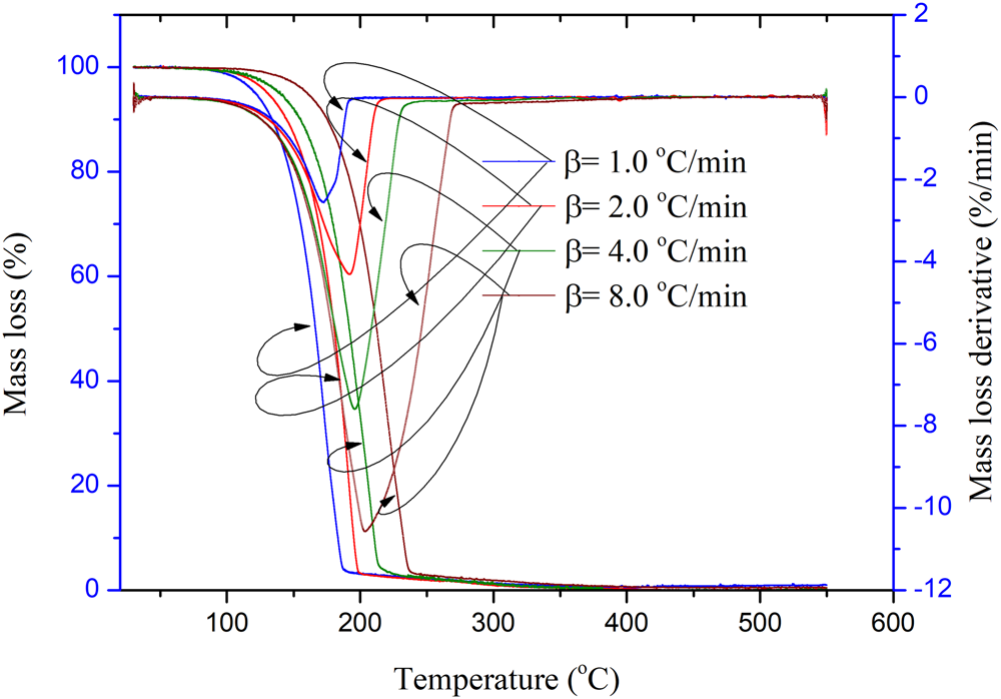
TG and DTG experimental results of EEC under prominent UV radiation for two months at four heating rates of 1.0, 2.0, 4.0, and 8.0 °C/min.

TG was applied to determine the thermal decomposition characteristics of EEC and EEC with UV under different durations. Figures 1–3 illustrate the experimental results of EEC and EEC under prominent UV radiations for different durations. It can be observed that when the heating rate increased, the initial temperature of mass loss was gradually delayed. These were the standard phenomena of the TG test when the heating rates increased progressively^26–29^. From Fig. 1, it can be seen that the average apparent onset temperature to evaluate the thermal decomposition characteristics of EEC, and EEC’s average apparent onset temperature was ca. 180.0 °C. In addition, the average peak temperature of EEC was 212.6 °C; the EEC’s characteristic parameters for the thermal decomposition of each heating rate are given in Table 2.

**Table 2:** Characteristic parameters of the thermal decomposition in TG tests for EEC at four heating rates of 1.0, 2.0, 4.0, and 8.0 °C/min.

β (°C/min)	*T*_0_ (°C)	*T*_p_ (°C)	*T*_f_ (°C)	Mass loss derivative (%/min)
1.0	176.3	187.6	195.9	−3.6
2.0	169.0	203.6	215.6	−5.4
4.0	181.3	220.2	224.7	−11.0
8.0	193.2	238.8	249.8	−16.8

From Figs 2 and 3, when EEC was under intensive UV at one and two months, respectively, the average apparent onset temperatures of EEC appeared in advance. The value of the average apparent onset temperatures of EEC under intensive UV at one and two months were 175.3 and 150.1 °C, respectively. The peak temperatures of EEC under prominent UV at one and two months were also 207.1 and 190.8 °C. Hence, the peak temperatures of EEC sustained with intensive UV had the same tendency; which means that thermal decomposition characteristics of EEC under intensive UV had increased. Hence, the authors confirmed that the effects of UV on EEC might result in the increasing and aging of thermal decomposition characteristics of EEC. Characteristic parameters of the thermal decomposition of EEC under prominent UV for the different durations are listed in Tables 3 and 4.

**Table 3:** Characteristic parameters of the thermal decomposition in TG tests for EEC under prominent UV radiation for one months at four heating rates of 1.0, 2.0, 4.0, and 8.0 °C/min.

β (°C/min)	*T*_0_ (°C)	*T*_p_ (°C)	*T*_f_ (°C)	Mass loss derivative (%/min)
1.0	144.8	168.6	186.9	−2.4
2.0	175.6	203.1	213.7	−6.0
4.0	189.2	222.0	231.5	−11.1
8.0	191.4	234.8	253.1	−17.4

**Table 4:** Characteristic parameters of the thermal decomposition in TG tests for EEC under prominent UV radiation for two months at four heating rates of 1.0, 2.0, 4.0, and 8.0 °C/min.

β (°C/min)	*T*_0_ (°C)	*T*_p_ (°C)	*T*_f_ (°C)	Mass loss derivative (%/min)
1.0	132.3	168.2	195.2	−2.5
2.0	154.9	184.5	209.3	−4.3
4.0	144.1	197.0	220.4	−7.6
8.0	169.2	213.3	240.2	−10.6

### Experiments by Differential Scanning Calorimetry

**Figure 4:**
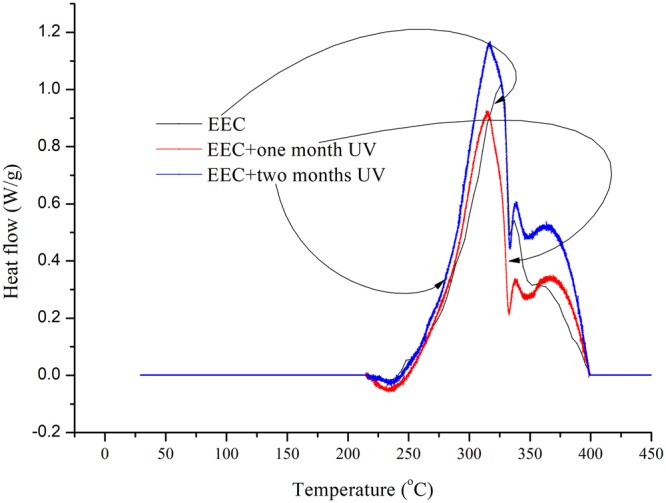
DSC experiments of EEC and EEC under prominent UV radiation for one and two months at 1.0 °C/min.

We selected DSC^30^ to investigate the heat production of EEC and EEC under prominent UV for one and two months. Figure 4 shows the experimental results of EEC and EEC irradiated with UV for one and two months. It was noticed that the tendency of onset and peak temperatures from the three samples were all the same with TG results at heating rate of 1.0 °C/min. The three curves from DSC results were compared and it was observed that there exist a number of peaks after EEC sustained with UV for one and two months. However, we could not find this phenomenon in the DSC results from pure EEC test. Hence, it was dangerous when the temperature of the entire reaction which belonged to EEC under prominent UV for one and two months was higher than the apparent onset temperature. Then, comparing the DSC results between EEC and EEC under prominent UV for one and two months, the temperature of the entire reaction was higher than 350.0 °C. The exothermic reaction continued and the reaction’s intensity was enhanced, which might result in a severe crisis.

Furthermore, it was observed that the heat generation from EEC and EEC stayed with UV for one and two months, and the value of EEC and EEC recorded from UV irradiation for one and two months was 664.0, 600.1, and 912.0 J/g, respectively.

Therefore, EEC irradiation with UV for two months had higher potential hazard in the analysis of the DSC experiments. Accordingly, it was noticed that EEC under prominent UV had several stages in the reaction and released more exothermic heat in the last stage of the reaction than pure EEC. Finally, it is better to avoid exposing EEC to UV for extended periods of time. Table 5 presents the process thermal safety and decomposition parameters of EEC and EEC under prominent UV with one and two months.

**Table 5:** Characteristic parameters of the thermal decomposition by DSC tests for EEC and EEC stayed for one and two months by UV at heating rate of 1.0 °C/min.

Sample	*T*_0_ (°C)	*T*_p_ (°C)	*T*_f_ (°C)	Δ*H*_d_ (kJ/kg)
EEC	252.3	326.3	391.7	664.0
EEC + one month UV	249.4	316.1	394.9	600.1
EEC + two months UV	244.7	317.0	399.8	912.0

### Evaluation of Thermokinetic Parameters by Dynamic Analysis

Four thermokinetic models were applied^31–39^ to understand the thermokinetic parameters for EEC undergoing with UV for different durations.

First, Flynn-Wall-Ozawa (FWO) model^33^, a common kinetic model which is very effective to describe the phenomena of reaction, was chosen to determine the *E*_a_ of EEC after prolonged exposure to UV. From FWO, Eqs (1–4) were generated as illustrated below:

1 $$G(\alpha )=\frac{A(\alpha )}{\beta }{{\rm{\int }}}_{{\rm{0}}}^{{\rm{T}}}\exp (\frac{-E{\rm{a}}}{R{T}_{{\rm{\alpha }}}}){\rm{d}}T=\frac{A(\alpha )E{\rm{a}}}{\beta R}{{\rm{\int }}}_{\infty }^{u}\frac{-{e}^{-u}}{{u}^{2}}{\rm{d}}u=\frac{AE{\rm{a}}}{\beta R}p(u)$$

2 $$\mathrm{ln}\,G(\alpha )=\,\mathrm{ln}(\frac{A(\alpha )E{\rm{a}}}{R})-\,\mathrm{ln}(\beta )+\,\mathrm{ln}(p(u))$$

3 $$p(u)=\frac{\exp (\,-\,u)}{u}-{\int }_{-\infty }^{u}\frac{\exp (\,-\,u)}{u}\,{\rm{d}}z$$

**Figure 5:**
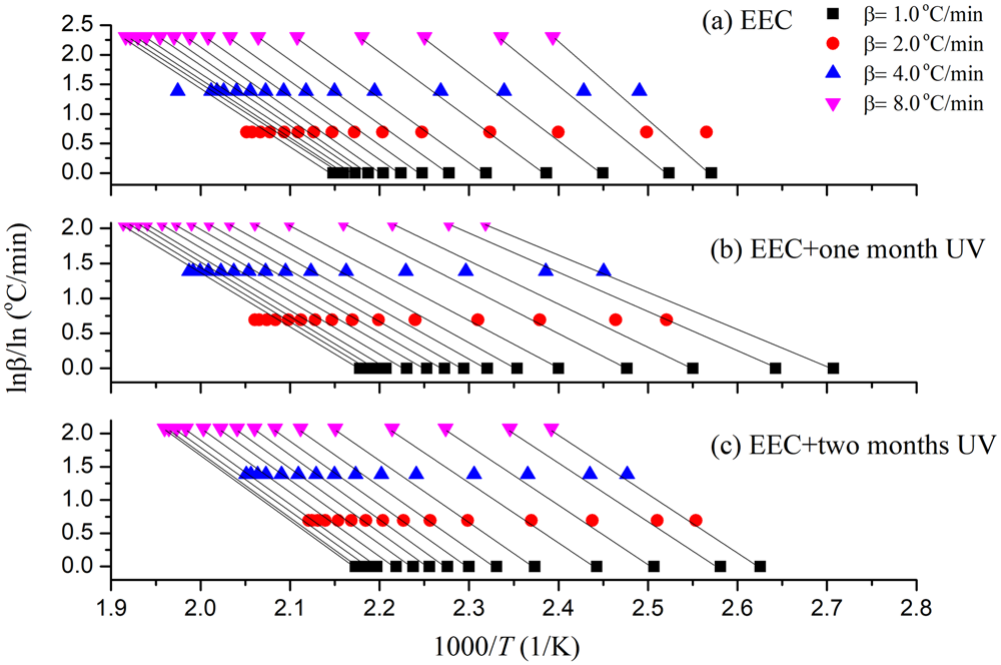
Simulation results for EEC and EEC under prominent UV radiation for one and two months through FWO model.

**Figure 6:**
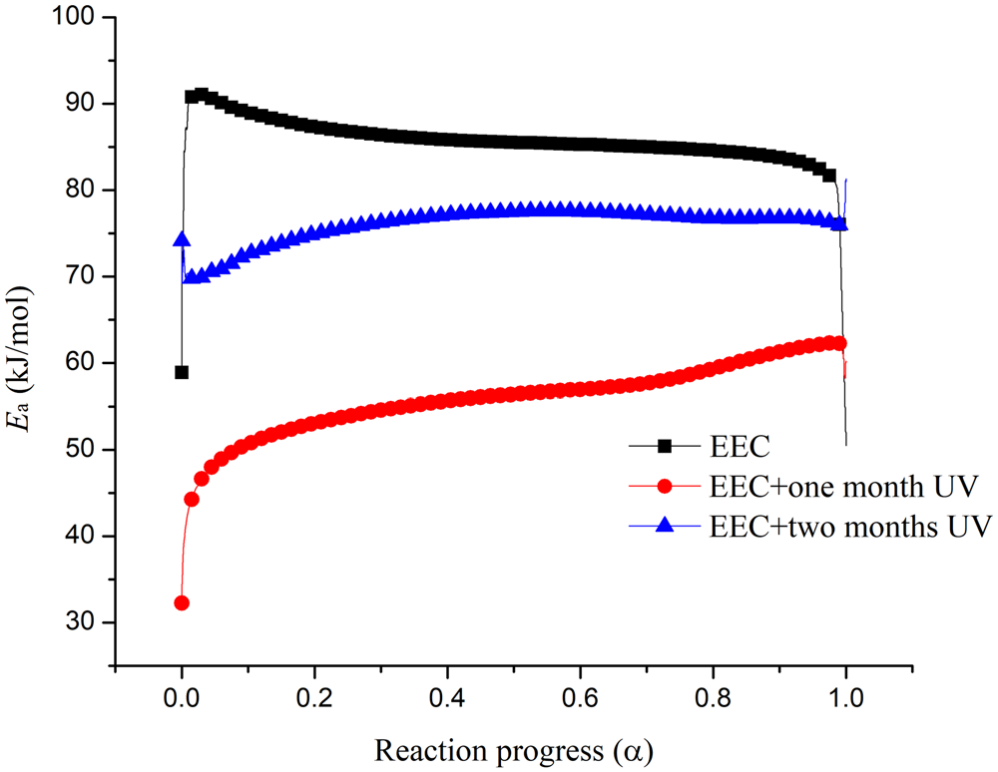
Calculated of *E*_a_ versus α for EEC and EEC under prominent UV radiation for one and two months by FWO model.

Then, *p*(*u*), which is temperature integral function, had an approximate solution through Doyle formula, and the constant of Eq. (4) could be obtained:

4 $$\mathrm{ln}(\beta )=\,\mathrm{ln}(\frac{A(\alpha )E{\rm{a}}}{RG(\alpha )})-5.331-1.052\frac{E{\rm{a}}}{R{T}_{{\rm{a}}}}$$

where *A*(α) is pre-exponential factor, *β* is the different heating rate (°C/min), *E*_a_ can be represented as apparent activation energy (kJ/mol), *G*(α) is integral form of the mechanism function, *T* is the temperature of conversion, and *R* is the universal gas constant (8.314 J/(mol K)). In Eq. (4), the same conversion of *E*_a_ is a fixed value, and the right-hand side of the equation first is a certain value. We fitted ln *β* and 1/*T*_a_ to calculate *E*_a_ and subsequently used conversion *α* to reckon the corresponding *E*_a_. The FWO results of EEC and EEC undergoing with different durations at one and two months by UV are shown in Fig. 5(a–c). Figure 6 illustrates the calculation of *E*_a_ through FWO model for EEC and EEC irradiated for one and two months with UV. From the literature review^34,35^, we determined that α of the main reaction in TG test was between 0.2–0.8. Therefore, the range of *E*_a_ in the main reaction (α = 0.2 − 0.8) of EEC and EEC under different durations of UV at one and two months was 84.53–87.33, 53.06–59.34, and 74.93–77.59 kJ/mol, respectively. The results showed that EEC irradiated for one month by UV had lower *E*_a_ value than other conditions.

Second, Friedman model, a method derived from a differential equation, was employed for understanding the whole reaction. Accordingly, the model is shown in Eq. (5):

5 $$\mathrm{ln}\,\frac{{\rm{d}}{\alpha }}{{\rm{d}}t}=\,\mathrm{ln}[A(\alpha )f(\alpha )]-(\frac{E{\rm{a}}}{RT})$$

**Figure 7:**
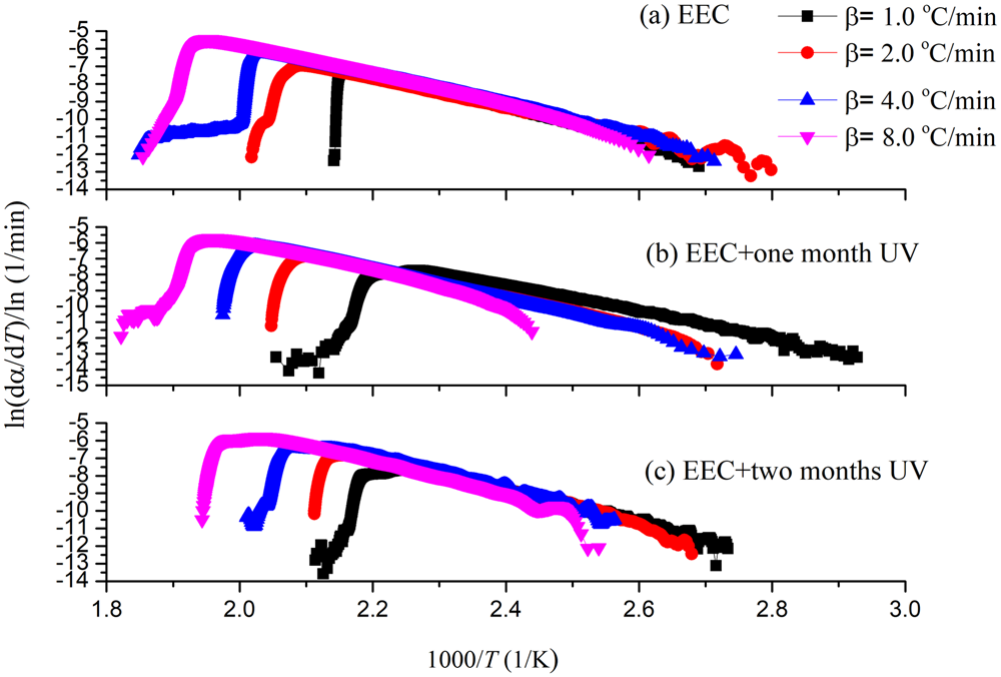
Simulation results on ln(dα/d*T*) versus 1/*T* for EEC and EEC under prominent UV radiation for one and two months through Friedman model.

**Figure 8:**
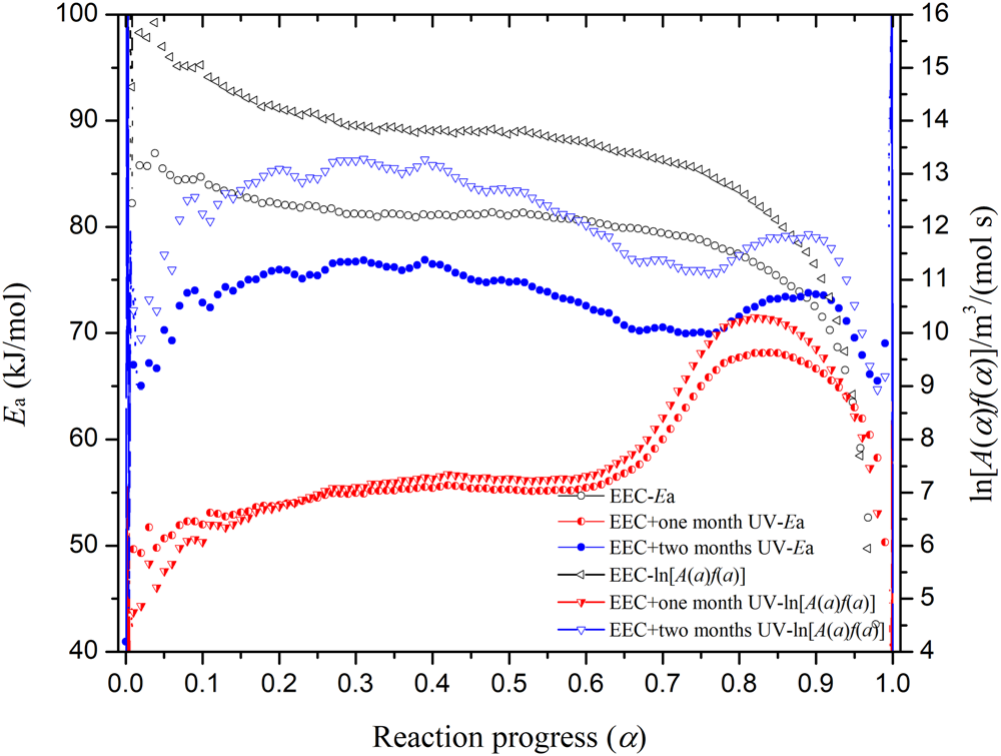
Calculated *E*_a_ and ln[*A*(α)*f*(α)] versus α for EEC and EEC and EEC under prominent UV radiation for one and two months by Friedman model.

From Friedman model^32,33^, the TG data could be analyzed from the natural logarithm of the conversion rate, dα/d*t*, which means that it is a function of the reciprocal absolute temperature (T) for transformation. Figure 7 shows the Friedman results of EEC and EEC under prominent UV for one and two months. From the calculation of *E*_a_ and ln[*A*(α)*f*(α)] in Fig. 8, the tendency of *E*_a_ for the entire reaction was the same with the tendency of *E*_a_ through FWO model. The scope of *E*_a_ in the main reaction (α = 0.2 – 0.8) of EEC and EEC sustained in different durations of UV at one and two months was 77.40–82.47, 53.83–67.66, and 77.62–82.09 kJ/mol, respectively. In addition, the area of ln[*A*(α)*f*(α)] in the major reaction of EEC and EEC under different durations of UV at one and two months was 12.68–14.32, 6.73–10.22, and 11.13−13.22 m^3^/(mol s), respectively.

From Fig. 8, ln[*A*(α)*f*(α)] of EEC has not much difference; therefore, it was a relatively stable reaction. However, when EEC was irradiated with UV for one month, ln[*A*(α)*f*(α)] increased with α in the severe reaction. The range of ln[*A*(α)*f*(α)] of EEC under one month was larger than other samples. Accordingly, it indicated that the scenario of EEC under prominent UV irradiation was thermally unstable. In addition, *E*_a_ value of EEC under prominent UV with one month was much lower than EEC and EEC under prominent UV with two months. Furthermore, the results of Friedman and FWO models pointed out that *E*_a_ value of EEC under prominent UV with one month was less than the others. This also showed that EEC under prominent UV with one month had higher potential hazard than pure EEC.

Third, the American Society for Testing and Materials (ASTM)^36^ has provided special methods, and one of these methods, E698–5, could be used to calculate *E*_a_ and *A* could be acquired by thermokinetic model. The formula is expressed in Eq. (6) ^33^:

6 $$\frac{{\rm{d}}{\alpha }}{{\rm{d}}t}=\frac{{\rm{A}}}{\beta }\exp (\frac{-E{\rm{a}}}{RT})\,f(\alpha )$$

**Figure 9:**
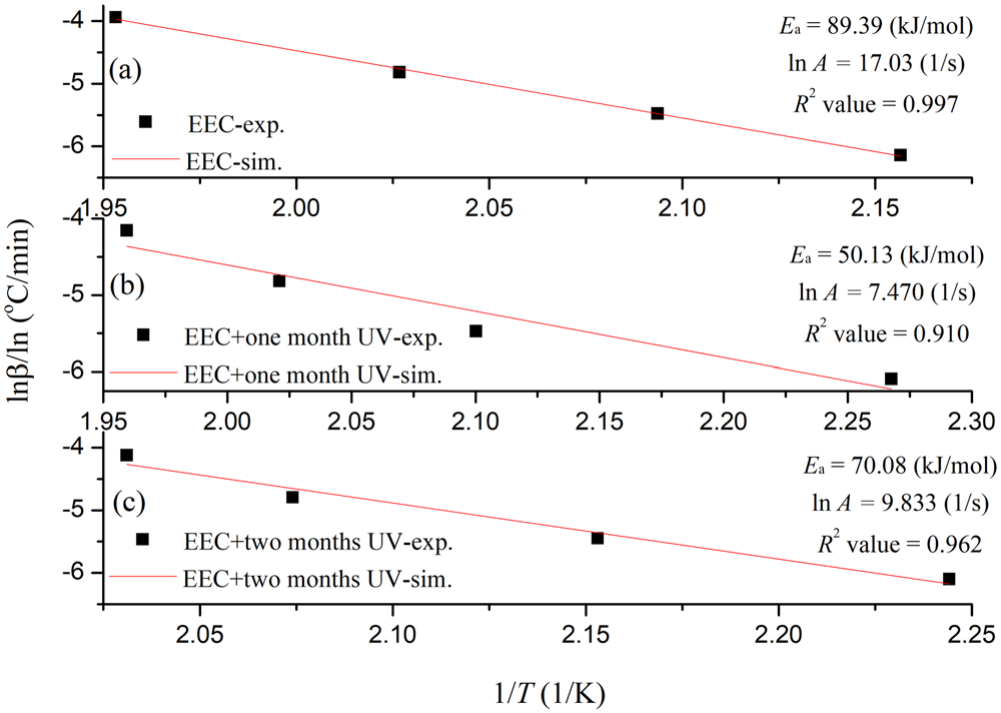
*E*_a_ and *A* calculation for EEC and EEC under prominent UV radiation for one and two months by ASTM E698-5.

This method involves linear regression at a chosen heating rate from the natural logarithm of the heating rate (ln*β*) plotted against 1/*T* that could obtain *E*_a_ and *A*, which were determined based on the slope (−*E*_a_*/R*) and intercept, respectively. Figure 9 shows the *E*_a_ and ln*A* calculation results for EEC and EEC under prominent UV radiation with one and two months. According to the experimental results, *E*_a_ of pure EEC and EEC under UV radiation with one and two months was 89.39, 47.73, and 70.08 kJ/mol, respectively. Moreover, the ln*A* of EEC and EEC irradiated for one and two months by UV was 17.03, 7.470, and 9.833 1/s, respectively.

**Figure 10:**
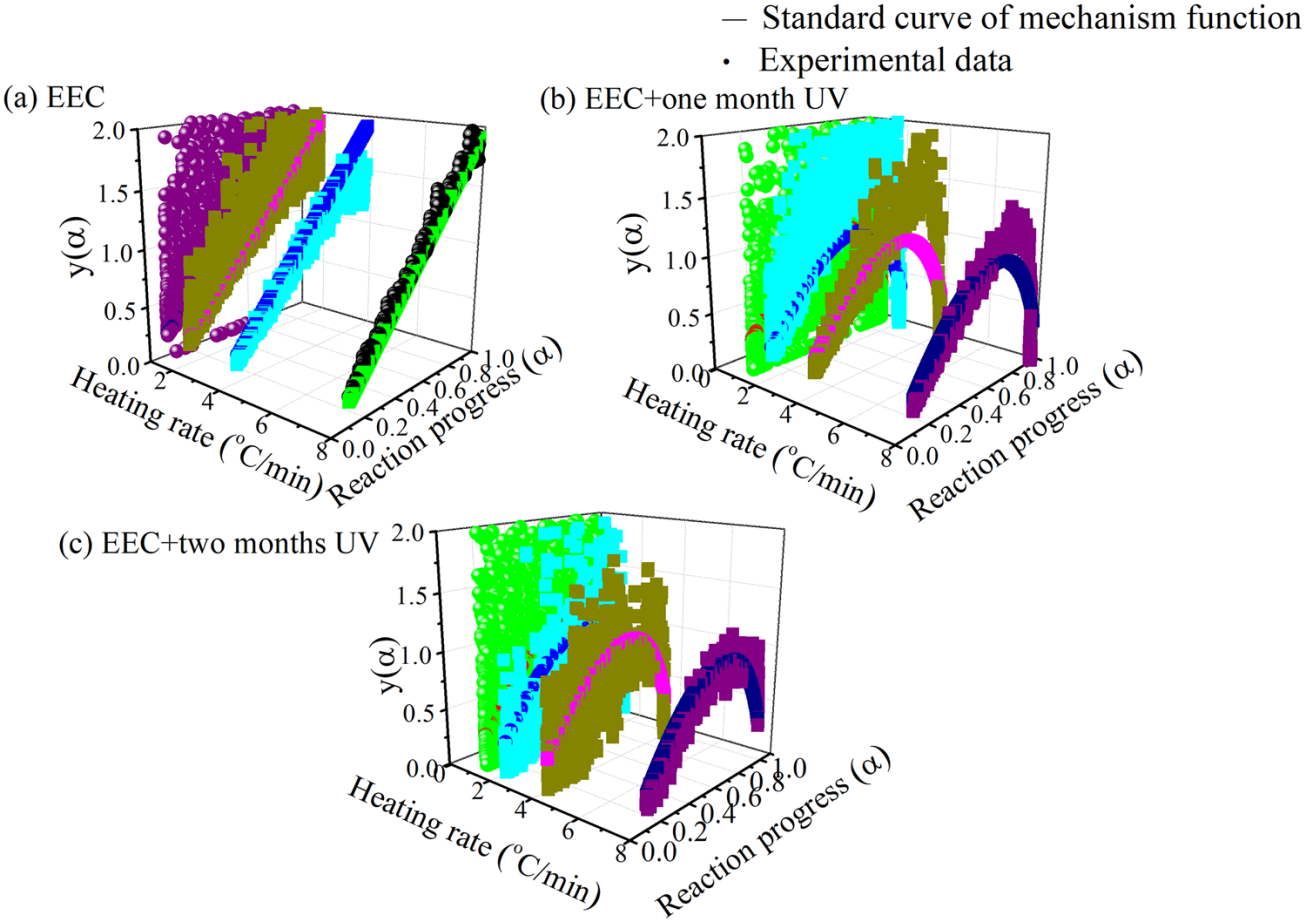
Comparison of experimental curves and standard curves for EEC and EEC under prominent UV with different durations at one and two months by Málek model.

Finally, the FWO and Friedman model are model-free methods, and the *f*(α) of the entire reaction cannot be obtained. Therefore, TG experimental scanned data for EEC and EEC under UV radiation with two durations of one and two months were used to explore the most probable kinetic function of the dominant decomposition process, through Málek model^37,38^. The Málek model could be demonstrated as shown in Eqs (7–9):

7 $${\rm{G}}(\alpha )=\frac{{\rm{R}}{T}^{{\rm{2}}}}{E{\rm{a}}\beta }\frac{{\rm{d}}\alpha }{{\rm{d}}t}\frac{1}{f(\alpha )}$$

8 $${\rm{G}}(0.5)=\frac{{\rm{R}}{T}_{0.5}^{2}}{E{\rm{a}}\beta }{(\frac{{\rm{d}}\alpha }{{\rm{d}}t})}^{0.5}\frac{1}{f(0.5)}$$

9 $${\rm{y}}(\alpha )=(\frac{T}{{T}_{0.5}})\frac{(\frac{{\rm{d}}\alpha }{{\rm{d}}t})}{{(\frac{{\rm{d}}\alpha }{{\rm{d}}t})}^{0.5}}=\frac{f(\alpha )G(\alpha )}{f(0.5)G(0.5)}$$

where y(α) is the defined function and f(α) is differential form of mechanism function of the reaction. In Eq. (9), the *y*(α) of different conversions could be determined by TG data. TG experimental curves for EEC and EEC were plotted under UV radiation with one and two months by *y*(α) and α. Then, the kinetic models were substituted into the Málek model to compare the resultant curve with the TG experimental curve, in order to validate which mechanism function that belongs to EEC and EEC under UV radiation with one and two months. The kinetic models of EEC and EEC under prominent UV with one and two months, expressed as *f*(α) = 0.67α^−0.5^, *G*(α) = α^1.5^ and *f*(α) = 2 (1−α) [−ln(1−α)]^0.5^, *G*(α) = [−ln(α)]^0.5^, respectively, were observed to have both high fitting degree and sound consistency. The heating rates of 1.0, 2.0, 4.0, and 8.0 °C/min for EEC and EEC sustained in UV under different durations of one and two months are drawn in Fig. 10.

According to the analysis by Málek model, it could be seen that the EEC’s decomposition form was not the same after irradiation, and thus chemical change might have occurred.

On the other hand, when EEC was under UV radiation for longer time, the decomposition pattern was not changed, and the reaction belonged to a physical change. In addition, the *E*_a_ of EEC and EEC under UV radiation with two durations of one and two months at different heating rates were calculated using Málek model, as presented in Table 6. From the calculation of the Málek model, the average *E*_a_ of EEC and EEC under UV radiation with two durations of one and two months was 95.50, 57.13, and 72.14 kJ/mol, respectively. According to the open literature, *E*_a_ of EEC in Málek model is similar to *E*_a_ which was provided in the reference^35^. Moreover, the results of EEC under prominent UV at different durations of one and two months were the same as the trends of *E*_a_ which were gained by the FWO, Friedman, and ASTM E698–5 models, and the *E*_a_ of EEC under prominent UV radiation for one month was the lowest among all the other samples.

**Table 6:** Calculated *E*_a_ for EEC and EEC under prominent UV radiation for one and two months at four heating rates through Málek model.

*E*_a_ (kJ/mol)	1.0 (°C/min)	2.0 (°C/min)	4.0 (°C/min)	8.0 (°C/min)	Average
EEC	91.22	92.32	99.31	99.16	95.50
EEC + one month UV	56.65	55.89	59.16	56.84	57.13
EEC + two months UV	77.93	79.29	70.98	60.36	72.14

### Component Analysis by GC-MS

**Figure 11:**
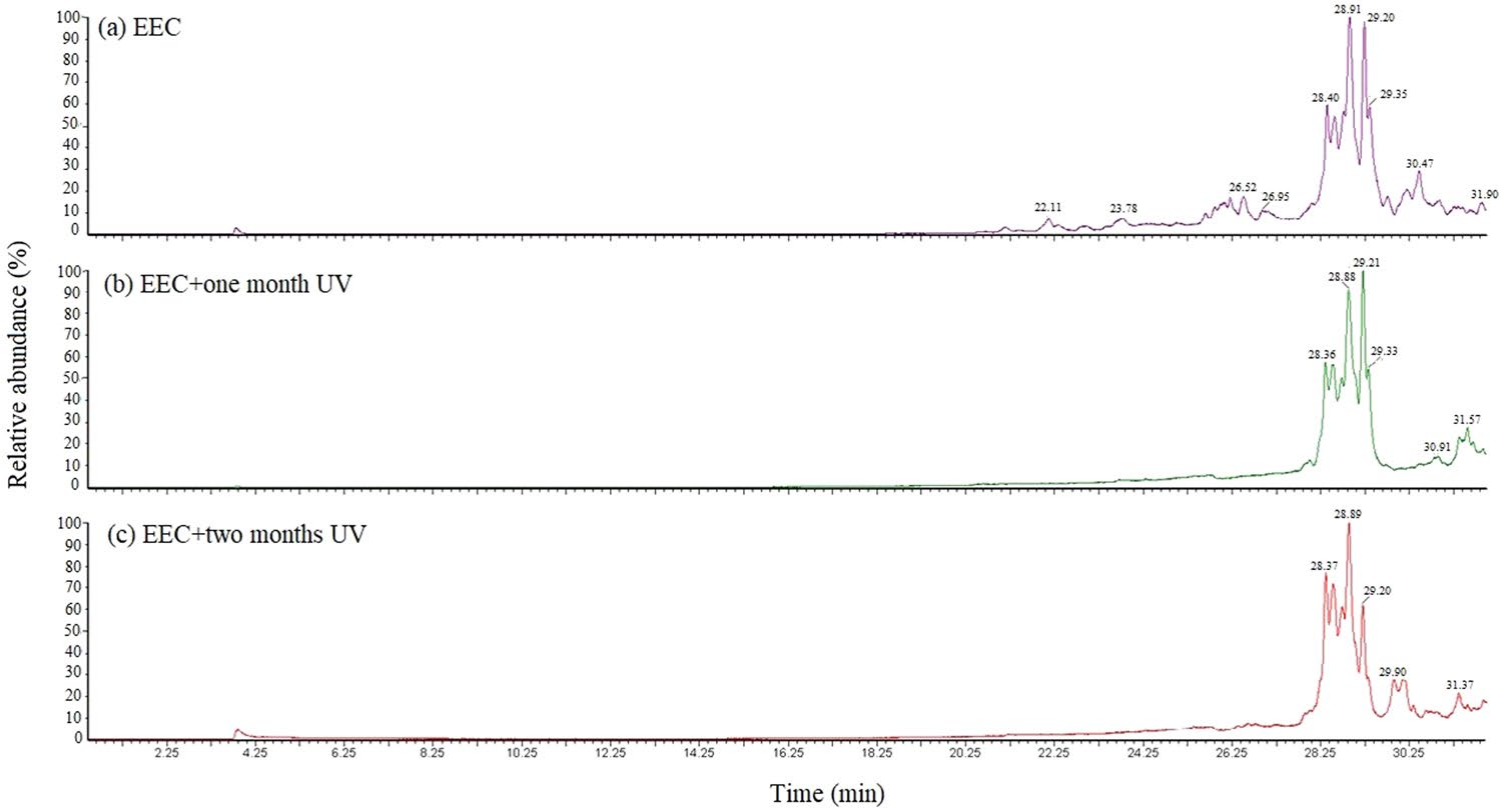
Decomposition compounds for EEC and EEC under prominent UV radiation for one and two months through GC-MS.

Figure 11 shows the GC data of EEC and EEC under prominent UV radiation for one month and two months. Table 7 describes the decomposition compound of EEC and EEC under prominent UV radiation for one month and two months from GC-MS. The main products of EEC were concentrated in 22.11–30.47 min, while the main products of EEC under prominent UV radiation for one month and two months were concentrated at 28.36–31.57 and 28.37–31.37 min, respectively. Compared with the product appearance time, when EEC was under prominent UV radiation, the products disappeared before 28.40 min in the pure EEC diagram. New substances were detected at 30.91 and 29.90 min in the GC diagram of one month and two months, respectively. The new substances belonged to a long carbon chain. From the above two points, it could be found that the detecting time and type of pure EEC experimental products were different from the products and detecting time after EEC illumination. The results of the experiment could be compared with the Málek model, and it was consistent with the function of reaction mechanism. It was feasible to use the Málek model function of reaction mechanism to indicate the difference in reaction.

**Table 7:** Components detected of EEC and EEC under prominent UV radiation for one and two months by GC–MS.

EEC		EEC + one month UV		EEC + two months UV	
Run time (min)	Name	Run time (min)	Name	Run time (min)	Name
22.11	3-Epoxyethyl-7-oxabicyclo[4.1.0]heptane	22.11	Non-detected	22.11	Non-detected
23.78	1,6,9-Tetradecatriene	23.78	Non-detected	23.78	Non-detected
26.52	3,4-Epoxycyclohexane methyl-3′4′-epoxycyclohexyl-carboxylate	26.52	Non-detected	26.52	Non-detected
26.95	Pentanal, 5-(methylenecyclopropyl)-	26.95	Non-detected	26.95	Non-detected
28.40	3,4-Epoxycyclohexane methyl-3′4′-epoxycyclohexyl-carboxylate	28.36	3,4-Epoxycyclohexane methyl-3′4′-epoxycyclohexyl-carboxylate	28.37	3,4-Epoxycyclohexane methyl-3′4′-epoxycyclohexyl-carboxylate
28.91	3,4-Epoxycyclohexane methyl-3′4′-epoxycyclohexyl-carboxylate	28.88	3,4-Epoxycyclohexane methyl-3′4′-epoxycyclohexyl-carboxylate	28.89	3,4-Epoxycyclohexane methyl-3′4′-epoxycyclohexyl-carboxylate
29.24	3,4-Epoxycyclohexane methyl-3′4′-epoxycyclohexyl-carboxylate	29.21	3,4-Epoxycyclohexane methyl-3′4′-epoxycyclohexyl-carboxylate	29.20	3,4-Epoxycyclohexane methyl-3′4′-epoxycyclohexyl-carboxylate
29.35	3,4-Epoxycyclohexane methyl-3′4′-epoxycyclohexyl-carboxylate	29.33	3,4-Epoxycyclohexane methyl-3′4′-epoxycyclohexyl-carboxylate	29.33	Non-detected
30.47	3,4-Epoxycyclohexane methyl-3′4′-epoxycyclohexyl-carboxylate	30.91	Dihomo-γ-linolenic acid	29.90	2-Pentadecynyl alcohol
31.90	3,4-Epoxycyclohexane methyl-3′4′-epoxycyclohexyl-carboxylate	31.57	3,4-Epoxycyclohexane methyl-3′4′-epoxycyclohexyl-carboxylate	31.37	3,4-Epoxycyclohexane methyl-3′4′-epoxycyclohexyl-carboxylate

## Conclusions

TG and DSC were used to evaluate the thermal decomposition and display the different features of EEC and EEC under prominent UV radiation. From the TG results, when EEC was irradiated with UV for one and two months, the average apparent onset temperatures were 4.7 and 29.9 °C in advance than pure EEC, respectively. The results of TG showed that when EEC was under prominent UV radiation, the thermal decomposition characteristics increased. According to DSC experimental results, we found that UV could cause a reaction from one stage to several stages and release more exothermic heat in the last stage of the reaction than pure EEC. Finally, FWO, Friedman, ASTM E698–5, and Málek models were provided to receive thermokinetic parameters for EEC and EEC under prominent UV, such as *A*, *E*_a_, and *f*(α) to evaluate the thermal behavior of EEC and EEC under prominent UV radiation. From the Málek model and DSC results, the *f*(α) of EEC’s decomposition pattern was not the same after irradiation, and it was also observed that the reaction peaks of EEC and EEC irradiated with UV for one and two months in the DSC experimental results were different (from only one exothermic peak to three exothermic peaks). On the other hand, the *E*_a_ of EEC under prominent UV for one month had the lowest value. Finally, from GC-MS results, the components showed the same tendency as the calculation of Málek model. This result illustrated that when EEC was under prominent UV radiation for long time in the event of high temperature and fire accidents, it may lead to serious thermal disasters.
